# Functional profile of a novel modulator of serotonin, dopamine, and glutamate neurotransmission

**DOI:** 10.1007/s00213-014-3704-1

**Published:** 2014-08-15

**Authors:** Gretchen L. Snyder, Kimberly E. Vanover, Hongwen Zhu, Diane B. Miller, James P. O’Callaghan, John Tomesch, Peng Li, Qiang Zhang, Vaishnav Krishnan, Joseph P. Hendrick, Eric J. Nestler, Robert E. Davis, Lawrence P. Wennogle, Sharon Mates

**Affiliations:** 1 Intra-Cellular Therapies, Inc., 3960 Broadway, New York, NY 10032 USA; 2 Centers for Disease Control and Prevention, National Institute for Occupational Safety and Health (CDC-NIOSH), Morgantown, WV 26505 USA; 3 Mount Sinai School of Medicine, New York, NY 10028 USA; 4 3D-Pharmaceutical Partners, San Diego, CA 92130 USA; 5 Present Address: Beth Israel Deaconess Medical Center, Boston, MA 02215 USA

**Keywords:** Schizophrenia, Dopamine D_2_ receptor, NMDA receptors, Serotonin 5-HT_2A_ receptor, Social defeat, Nucleus accumbens, Microdialysis, Mesocortical, Nigrostriatal, Serotonin transporter

## Abstract

**Rationale:**

Schizophrenia remains among the most prevalent neuropsychiatric disorders, and current treatment options are accompanied by unwanted side effects. New treatments that better address core features of the disease with minimal side effects are needed.

**Objectives:**

As a new therapeutic approach, 1-(4-fluoro-phenyl)-4-((6bR, 10aS)-3-methyl-2,3,6b,9,10,10a-hexahydro-1H,7H-pyrido[3′,4′:4,5]pyrrolo[1,2,3-de]quinoxalin-8-yl)-butan-1-one (ITI-007) is currently in human clinical trials for the treatment of schizophrenia. Here, we characterize the preclinical functional activity of ITI-007.

**Results:**

ITI-007 is a potent 5-HT_2A_ receptor ligand (*K*_*i*_ = 0.5 nM) with strong affinity for dopamine (DA) D_2_ receptors (*K*_*i*_ = 32 nM) and the serotonin transporter (SERT) (*K*_*i*_ = 62 nM) but negligible binding to receptors (e.g., H_1_ histaminergic, 5-HT_2C_, and muscarinic) associated with cognitive and metabolic side effects of antipsychotic drugs. In vivo it is a 5-HT_2A_ antagonist, blocking (±)-2,5-dimethoxy-4-iodoamphetamine hydrochloride (DOI)-induced headtwitch in mice with an inhibitory dose 50 (ID_50_) = 0.09 mg/kg, per oral (p.o.), and has dual properties at D_2_ receptors, acting as a postsynaptic D_2_ receptor antagonist to block D-amphetamine hydrochloride (D-AMPH) hyperlocomotion (ID_50_ = 0.95 mg/kg, p.o.), yet acting as a partial agonist at presynaptic striatal D2 receptors in assays measuring striatal DA neurotransmission. Further, in microdialysis studies, this compound significantly and preferentially enhances mesocortical DA release. At doses relevant for antipsychotic activity in rodents, ITI-007 has no demonstrable cataleptogenic activity. ITI-007 indirectly modulates glutamatergic neurotransmission by increasing phosphorylation of GluN2B-type *N*-methyl-d-aspartate (NMDA) receptors and preferentially increases phosphorylation of glycogen synthase kinase 3β (GSK-3β) in mesolimbic/mesocortical dopamine systems.

**Conclusion:**

The combination of in vitro and in vivo activities of this compound support its development for the treatment of schizophrenia and other psychiatric and neurologic disorders.

## Introduction

Schizophrenia is a major neuropsychiatric disorder that affects over 1 % of the world’s population. It is characterized by the experience of hallucinations and delusions, referred to as “positive” symptoms, and by a variety of other symptoms, including decreased social function and speech, flat affect, disorganized thought, and low motivation, referred to as “negative” symptoms. Cognitive impairment is also a core feature of schizophrenia. The treatment of schizophrenia was revolutionized 60 years ago with the introduction of the first generation of antipsychotic medications, now referred to as “typical” antipsychotic medications, which have in common the ability to strongly inhibit activity of dopamine receptors of the D_2_ subclass (Creese et al. 1976). Typical antipsychotic drugs are effective treatments for reducing positive symptoms in many patients. Owing to their potent D_2_ receptor antagonism within the nigrostriatal motor system, their utility is limited by severe motor abnormalities including acute parkinsonian movement deficits and dystonia, referred to generally as extrapyramidal motor syndromes (EPS), and drug-induced tardive dyskinesia.

A newer generation of antipsychotic medications, called “atypical” antipsychotics, combines potent inhibition of serotonin-2A (i.e., 5-HT_2A_) receptors with dopamine D_2_ receptor antagonism (Meltzer et al. 1989). All of the atypical drugs exhibit a relatively high-affinity inhibition of the serotonin 5-HT_2A_ receptor (Meltzer et al. 1989), supporting the significance of this particular mechanism to the treatment of schizophrenia. These atypical drugs offer an improved side effect profile with regard to motor function and treatment-resistant psychosis relative to first-generation antipsychotics (Kane et al. 1988). Unfortunately, enthusiasm for the newer generation of antipsychotic medications has been tempered by the emergence of other severe and often debilitating side effects, including a liability for profound weight gain (as much as 50 lbs/year), an increased incidence of type II diabetes, cognitive impairment, sedation, orthostatic hypotension, blurred vision, constipation, dizziness, and loss of bladder control. The side effects appear to be associated with non-selective interactions of these medications with receptors that are unrelated to antipsychotic efficacy, including serotonergic 5-HT_2C_, histaminergic H_1_, alpha-adrenergic, and muscarinic receptors (Kroeze et al. 2003; Matsui-Sakata et al. 2005; Lieberman et al. 2005; Nasrallah 2008). An antipsychotic agent having potent effects at 5-HT_2A_ receptors that subserve antipsychotic efficacy, yet minimal interactions with specific receptor subclasses associated with these side effect liabilities would represent a significant advance in the safe and effective treatment of schizophrenia and the quality of life of patients.

The effectiveness of D_2_ receptor antagonists in addressing positive symptoms supports a key role for abnormal dopamine neurotransmission in the etiology of schizophrenia (Davis et al. 1991). Current antipsychotic medications, though, fail to substantially improve negative features of the disease. Moreover, these medications can further compromise poor cognitive function in schizophrenic patients (Keefe et al. 2007). Clearly, abnormalities in brain neurotransmitters in addition to dopamine figure prominently in the complex features of the disease. In particular, various types of data indicate that glutamate neurotransmission mediated through *N*-methyl-d-aspartate (NMDA)-type receptors, present in brain areas involved in cognition, is deficient in schizophrenic patients (Javitt 2007). Deficits in glutamatergic neurotransmission in cerebral cortex likely contribute to hyperdopaminergic activity in subcortical regions, responsible for positive symptoms of schizophrenia (Laruelle et al. 2005). The data also implicate deficits in glutamate neurotransmission in the etiology of negative features of schizophrenia and cognitive impairments. A new antipsychotic medication acting synergistically via serotonergic, dopaminergic, and glutamatergic systems and able to benefit the social functioning of patients while addressing positive symptoms of the disease without inducing extrapyramidal symptoms (EPS) would be of tremendous benefit to schizophrenic patients.

Here, we describe the biochemical and behavioral characterization of 1-(4-fluoro-phenyl)-4-((6bR, 10aS)-3-methyl-2,3,6b,9,10,10a-hexahydro-1H,7H-pyrido[3′,4′:4,5]pyrrolo[1,2,3-de]quinoxalin-8-yl)-butan-1-one (ITI-007), a tosylate salt, as a novel small-molecule therapeutic agent displaying the combined properties of potent 5-HT_2A_ antagonism, cell type-specific modulation of dopamine protein phosphorylation pathways, and serotonin transporter binding, currently in development for the treatment of schizophrenia.

## Materials and methods

### Materials

ITI-007 (MW 565.7), a tosylate salt, was synthesized at Intra-Cellular Therapies, Inc. Haloperidol, clozapine, D-amphetamine, and (±)-2, 5-dimethoxy-4-iodoamphetamine hydrochloride (DOI) were obtained from Sigma-Aldrich Chemical Co. (St. Louis, MO). Risperidone, olanzapine, quetiapine, and aripiprazole were obtained from Toronto Research Chemicals, Inc. (Toronto, Canada). All radioligands used for receptor binding studies were obtained from New England Nuclear (Boston, MA).

### In vitro binding affinity

Binding activity at a variety of receptor targets, including recombinant human serotonin receptors and rat dopamine D_2_ and human recombinant dopamine D_4_ receptors, was measured in vitro using standard radioligand displacement methods (NovaScreen/Caliper/Perkin-Elmer, Hanover, MD), to determine affinity. Binding of ITI-007 at 5-HT_2A_ was measured at human recombinant receptors expressed in human embryonic kidney 293E (HEK293E) cells and using ^125^I-DOI as a radioligand and by binding in rat cortex using ^3^H-ketanserin as a radioligand. Functional activity of ITI-007 at 5-HT_2A_ receptors was studied by measuring serotonin-mediated increases in calcium fluorescence and confirmed by phosphatidylinositol (PI) turnover in HEK293E cells expressing the 5-HT_2A_ receptor, as described (Porter et al. 1999; Conn and Sanders-Bush 1984). D_2_ receptor binding was measured at rat recombinant receptors expressed in Chinese hamster ovary (CHO) cells and using ^3^H-methylspiperone as a radioligand and in rat striatum using ^3^H-sulpiride as a radioligand. Functional activity at D_2_ receptors was measured as blockade of dopamine-induced inhibition of forskolin-stimulated (10 μM) cAMP accumulation in CHO cells expressing human recombinant D_2_-short receptor. D_4_ receptor binding was measured at human recombinant receptors expressed in CHO cells and using ^3^H-methylspiperone as a radioligand. D_1_ receptor binding at human recombinant receptor was measured in CHO cells using ^3^H-SCH23390 as a radioligand. 5-HT_2C_ receptor binding was measured in pig choroid plexus using ^3^H-methylsergide as a radioligand. Binding to alpha adrenergic receptors in the rat frontal cortex (alpha_1A_) or rat liver (alpha_1B_) was measured using ^3^H-prazosin as a radioligand. Histamine H_1_ receptor binding was measured in bovine cerebellum using ^3^H-pyrilamine as a radioligand. Binding to the serotonin transporter (SERT) was measured in CHO cells expressing a human recombinant transporter using ^3^H-imipramine as a radioligand and confirmed against native transporter in rat forebrain synaptosomal membranes. The affinity constant (*K*_*i*_) for each receptor subtype or inhibitory concentration 50 (IC_50_) for inhibition in functional assays was calculated.

### In vitro binding in a broad selectivity panel

The in vitro binding of ITI-007 across a panel of over 60 neurotransmitter receptors, ion channels, neurotransmitter transporters, and synthetic enzymes was evaluated to investigate the selectivity of the compound with the NovaScreen SEP Broad Profile panel performed using a 100-nM concentration of the compound (NovaScreen/Caliper/Perkin-Elmer, Hanover, MD).

### Animals

Male C57BL/6J mice (7–8 weeks of age), obtained from The Jackson Laboratory (Bar Harbor, ME), were used for biochemical measures of TH, glycogen synthase kinase 3β (GSK-3β), and GluN2B phosphorylation state, catalepsy experiments, and determination of DOI-induced headtwitch (performed at ITI) and for the social defeat paradigm (performed at Mount Sinai School of Medicine). Male CD1 retired breeder mice were obtained from Charles River Laboratories (Wilmington, MA) and served as aggressors for the social defeat studies. Male Sprague–Dawley rats (150–200 g) were purchased from Charles River Laboratories (Wilmington, MA) for D-amphetamine hyperactivity studies (ITI) and for dopamine metabolism studies (Centers for Disease Control—National Institute for Occupational Safety and Health, CDC-NIOSH). Male Wistar rats (280–350 g) were obtained from Harlan Labs (Livermore, CA) for microdialysis studies contracted with BrainsOnline (Groningen, The Netherlands) and performed at the University of California at San Francisco. In all cases, animals were maintained in standard laboratory conditions under a 12-h light/dark cycle with food and water available ad libitum with a minimum of 1-week acclimation prior to experimentation. All experiments were carried out in accordance with the National Institute of Health Guide for the Care and Use of Laboratory Animals (NIH Publications No. 80–23) and with the approval of the Institutional Animal Care and Use Committees at the respective institutions and contract research organizations.

### Compound formulation for animal dosing

Unless otherwise indicated, ITI-007, aripiprazole, and risperidone were dissolved in solution of 0.5 % (*w*/*v*) methylcellulose (400 cP, #M0430, Sigma-Aldrich Chemical Co., Inc.) in water. Oral dosing solutions were prepared fresh daily. Haloperidol and clozapine were dissolved in a small volume of glacial acetic acid or 1 N HCl, which was further diluted with addition of either 2 % acetic acid in saline (0.9 % NaCl in water) or 0.1 N HCl in saline. The pH of the dosing solutions was adjusted to pH 5.5 by dropwise addition of 0.1 N NaOH in saline and volume adjusted by addition of saline. All other compounds were dissolved in physiological saline solution. ITI-007 was administered, per oral (p.o.) by gastric gavage to mice in a volume of 6.67 ml/kg body weight or to rats in a volume of 2 ml/kg body weight, unless otherwise stated. Routes of administration and injection volumes of other compounds were as indicated.

### Functional assessment of 5-HT_2A_ antagonism

The 5-HT_2A_ agonist, DOI, was used to elicit stereotyped headtwitch behavior in mice using a protocol modified from Gardell et al. (2007). Inhibition of DOI-induced headtwitch was measured as a functional assay for the in vivo potency of the compound as an antagonist at 5-HT_2A_ receptors. C57Bl/6 mice (8–10 weeks in age, *N* = 4/group) were injected with vehicle (saline) or with the 5-HT_2A_ agonist, DOI (2.5 mg/kg intraperitoneal (i.p.), in saline). Other mice were given an oral dose of ITI-007 (0.001–1 mg/kg in 0.5 % methylcellulose in water) 30 min prior to DOI. Headtwitches were then counted for 5 min, starting 10 min after DOI injection. An ID_50_ for inhibition of DOI-induced headtwitch was calculated using a four-parameter logistical fit (Excel Fit software, IDBS).

### Inhibition of amphetamine-induced hyperactivity compared with antipsychotic medications

Male Sprague–Dawley rats (200–250 g, *N* = 4/treatment group) were habituated to Lucite locomotor activity chambers comprising an AccuScan activity monitoring system (AccuScan Instruments, Inc., Columbus, OH) were maintained in a quiet observation room kept under low light conditions. Thirty minutes later, rats were given an oral dose of vehicle alone (0.5 % methylcellulose in water, 1 ml/kg volume) or vehicle containing a dose of ITI-007 (0.3–10 mg/kg), haloperidol (0.1–3 mg/kg), risperidone (0.3–3 mg/kg), or aripiprazole (1–30 mg/kg). The rats were briefly removed from the activity chambers 60 min later to receive an injection of D-amphetamine hydrochloride (D-AMPH; 1 mg/kg in 1 ml/kg, i.p.). The animals were returned to the activity chambers, and locomotor activity was then recorded for 2 h using system software. Activity was quantitated as distance traveled (cm) over 2 h for each group. Inhibitory activity was calculated as the percent of total activity observed at each dose level of each drug versus animals receiving D-AMPH alone. An ID_50_ for inhibition of D-AMPH-induced hyperactivity was calculated for each compound.

### Analysis of tyrosine hydroxylase phosphorylation state

Intracellular signaling effects of a panel of antipsychotic medications were analyzed in parallel with ITI-007 using the CNSProfile™ immunoblotting platform, as described previously (Zhu et al. 2010). The effect of the selected compounds on the phosphorylation state of tyrosine hydroxylase (TH) was measured and quantitated as an indicator of the potential of each compound to perturb presynaptic dopamine synthesis in striatum. For these studies, groups of mice (*N* = 6/time point) were treated with specified dose levels of each compound or with vehicle then killed 15, 30, or 60 min postinjection by focused microwave cranial irradiation (4.5 kW, 1.2-s duration) using a microwave applicator (Muromachi Kikai Ltd., Tokyo, Japan, model TMW-6402C). This technique preserves in vivo levels of brain protein phosphorylation (Zhu et al. 2010). Striatum was dissected from each mouse brain, frozen in liquid nitrogen, and stored at −80 °C until analyzed for phosphoprotein levels.

Frozen samples of brain tissue from microwave-irradiated mouse brains were sonicated in an aliquot of boiling 1 % (*w*/*v*) sodium dodecyl sulfate (SDS) and then boiled for an additional 10 min to further insure that postmortem kinase, phosphatase, and protease activities are inactivated. Small aliquots of the homogenate were retained for protein determination by the bicinchoninic acid (BCA) protein assay method (Pierce Chemical Co., Rockford, IL). Equal amounts of protein (15–50 μg) were processed by SDS/polyacrylamide gel electrophoresis (SDS-PAGE) using 10 % polyacrylamide gels and immunoblotted as described below and electrophoretically transferred to nitrocellulose membranes (BioRad, Hercules, CA). The membranes were blocked in a 50:50 mix of Tris-buffered saline (TBS 50 mM Tris–HCl, 150 mM NaCl, pH 7.5)/1 % (*v*/*v*) Tween 20 and LiCor Blocking Buffer (LiCor, Lincoln, NE). Immunoblotting was carried out using a phosphorylation state-specific polyclonal antibody raised against serine 40 (S40)-phosphorylated tyrosine hydroxylase (Chemicon, Temecula, CA) and a pan-TH monoclonal antibody for measuring the total protein levels (BD Biosciences, San Jose, CA). Membranes were washed four times for 5 min each with TBS/Tween 20, and antibody binding was detected using Alexa-680-labeled goat anti-mouse IgG (Molecular Probes, Eugene, OR) or IRdye-800CW-labeled goat anti-rabbit IgG (Rockland Immunochemicals, Gilbertsville, PA). Antibody binding was detected and quantitated using a LiCor Odyssey infrared fluorescent detection system.

Phosphorylation at each site detected by phospho-specific antibodies was quantified, normalized to total levels of the protein (non-phosphorylated), and expressed as percent ± SEM of the level of phosphorylation in vehicle-treated control mice (*N* = 6). Statistical analysis was performed using Student’s paired *t* test or ANOVA with Newman–Keuls post hoc test as indicated using GraphPad Prism 4.2 (GraphPad Software Inc., San Diego, CA), with *p* < 0.05 considered significant.

### Dopamine metabolism

The effects of haloperidol, risperidone, aripiprazole, or ITI-007 on striatal dopamine metabolism were compared in order to assess the comparative impact of these drug treatments on dopamine neurotransmission. Measurements of tissue dopamine levels were performed at CDC-NIOSH. Mice (*N* = 6/treatment group) were administered selected doses of ITI-007 (1, 3, or 10 mg/kg), haloperidol (1 or 3 mg/kg), risperidone (1 or 10 mg/kg), or aripiprazole (3 or 30 mg/kg) once or once daily p.o. for 21 days. Animals were killed by focused cranial microwave irradiation (4.0 kW, 0.9-s duration) using a microwave applicator (Muromachi Kikai, Ltd., Tokyo, Japan, model TMW-5012C) 2 h after the final dose of drug. Striatum was rapidly dissected from each mouse brain, weighed, and placed into Eppendorf tubes that were frozen in liquid nitrogen and stored at −80 °C.

Samples were analyzed using high-performance liquid chromatography with electrochemical detection (HPLC-EC) for levels of dopamine and dopamine metabolites 3,4-dihydroxyphenylacetic acid (DOPAC) and homovanillic acid (HVA). Tissues were homogenized in 100 μl of ice-cold 0.2 M perchloric acid containing 1 μM dihydroxybenzylamine (DHBA) as an internal standard and centrifuged at 10,000×*g* for 10 min at 4 °C. The supernatant was filtered through a 0.2 μm nitrocellulose membrane, and an aliquot (10 μl) was injected from a temperature-controlled (4 °C) automatic sample injector (Waters 717plus Autosampler) connected to a Waters 515 HPLC pump. Catecholamines were separated on a C_18_ reverse-phase column (LC-18 RP; Waters SYMMETRY 25 cm × 4.6 mm; 5 μm), electrochemically detected (Waters 464 Pulsed Electrochemical Detector; range 10 nA, potential +0.7 V), and analyzed using Millennium software. The mobile phase, pH = 3.0, for isocratic separation of dopamine consisted of dibasic sodium phosphate (75 mM), octane sulfonic acid (1.7 mM), acetonitrile (10 %, *v*/*v*), and ethylenediaminetetraacetic acid (EDTA) (25 μM). A flow rate of 1 ml/min was used. Dopamine, DOPAC, and HVA standards (0.5–25 pmol) were prepared in 0.2 M perchloric acid containing the internal standard (DHBA). The recovery of each analyte was adjusted with respect to the internal standard and quantified from a standard curve. The levels of dopamine and its metabolites, DOPAC and HVA, were calculated from the AUC values for each (based on microgram per gram (μg/g) tissue wet weight) and expressed as a percent of the saline control.

### Measurement of forelimb catalepsy in mice

The potential of ITI-007 for eliciting striatal motor side effects was evaluated using the mouse forelimb catalepsy model. Mice (*N* = 4/dose level) were administered ITI-007 (1, 3, 10, or 30 mg/kg, p.o.) or the D_2_ receptor antagonist, haloperidol (3 mg/kg, p.o.) or the vehicle solution (0.5 % methylcellulose in water) at time zero. Forelimb catalepsy was measured using the bar grip test. Mice were placed in a Lucite mouse cage outfitted with a 3-mm diameter wooden rod suspended 4 cm from the floor of the cage. During testing, the mouse was allowed to grip the bar firmly with hind legs placed firmly on the floor of the cage. The mouse was required to maintain this posture for at least 10 s for a valid test session. Catalepsy was then quantitated by recording the latency (in seconds) to step both front paws down to the floor of the cage up to a maximum time of 120 s. If the mouse stepped off the bar immediately (less than 10 s), another attempt was made up to a maximum of ten attempts. The longest duration of immobility was recorded if none of the ten attempts were beyond 10 s. Catalepsy was measured for each mouse at 2, 3, 4, and 6 h after drug administration. Mean forelimb catalepsy time (in seconds) was calculated across each group and time point. Data were analyzed using ANOVA with Newman–Keuls post hoc test.

### Measurement of dopamine in striatum and prefrontal cortex by in vivo microdialysis

Using in vivo microdialysis techniques performed at BrainsOnline (San Francisco, CA), the effect of ITI-007 on levels of extracellular dopamine in nigrostriatal (i.e., striatum) and mesocortical (i.e., prefrontal cortex) terminal regions was measured. Adult male Wistar rats (300–350 g) were prepared surgically for in vivo microdialysis with probes implanted in both prefrontal cortex (PFC) and striatum 24–48 h prior to the experiment. Rats were anesthetized for surgery using isoflurane (2 %, 800 ml/min O_2_), placed in a stereotaxic frame (Kopf Instruments), and I-shaped microdialysis probes (Hospal AN 69 membranes) inserted into the PFC and striatum (4 mm exposed surface for prefrontal cortex and 3 mm exposed for striatum). Coordinates used for placement of the probe tips were for the PFC (posterior (AP) = +3.4 mm to bregma, lateral (L) = −0.8 mm to midline, and ventral (V) = 6.0 mm to dura) and for the striatum (posterior (AP) = +0.9 mm to bregma, lateral (L) = −3.0 mm to midline, and ventral (V) = 5.0 mm to dura).

On the day of the experiment (24–48 h after surgery), the microdialysis probes of each animal were connected via the use of flexible PEEK tubing to a microperfusion pump (Harvard Apparatus, PHD 2000 Holliston, MA). Artificial CSF, composed of 147 mM NaCl, 3 mM KCl, 1.2 mM CaCl_2_, and 1.2 mM MgCl_2_ was used to perfuse the probes (1.5 μl/min) beginning at 75 min prior to drug treatment. Baseline dialysate collection was begun for all rats 60 min prior to drug administration (*t* = −60 min) such that a total of four baseline samples were collected prior to drug treatment (*t* = 0). At time *t* = 0 min, the rats received haloperidol (0.3 mg/kg dissolved in acidified water, s.c., *N* = 10), aripiprazole (30 mg/kg in 0.5 % methylcellulose in water, p.o., *N* = 5), or ITI-007 (3 or 10 mg/kg, p.o. in 0.5 % methylcellulose in water, *N* = 6/dose level). A control group of rats (*N* = 9) received only the 0.5 % methylcellulose vehicle solution, delivered orally (p.o.). All drug solutions were delivered in a volume of 1 ml/kg of body weight. Every 20 min for the following 3 h, dialysate was collected from both brain regions of each rat. Dialysates were collected into mini-vials containing 7.5 μl of 0.02 M formic acid using an automated fraction collector Univentor 820, Malta. Tubes containing dialysate samples were then frozen at −80 °C until analyzed by HPLC for levels of dopamine and the dopamine metabolite, DOPAC. After the final sample collection, all rats were killed and the brains placed for 3 days in a solution of 4 % (*w*/*v*) paraformaldehyde. Coronal sections were prepared of each rat brain to verify the position of the dialysis collection probes.

Dopamine and DOPAC concentrations were determined in each sample using HPLC separation and electrochemical detection. An aliquot (20 μl) of each dialysate sample was injected onto the HPLC column by means of a refrigerated microsampler system, consisting of a syringe pump (Gilson, model 402), a multi-column injector (Gilson, model 233 XL), and a temperature regulator (Gilson, model 832). The chromatographic separation was performed on a reverse-phase 150 × 2.1 mm (3 μm) C_18_ Thermo BDS Hypersil column (Keystone Scientific). The mobile phase (isocratic) consisted of a sodium acetate buffer (4.1 g/l) with methanol (2 %, *v*/*v*), EDTA (150 mg/l), octane sulfonic acid (180 mg/l), and tetramethylammonium chloride (150 mg/l), and the final solution adjusted to a pH = 4.1 using glacial acetic acid. The mobile phase was run at a flow rate of 0.35 ml/min using an HPLC pump (Shimadzu, model LC-10AD vp). Dopamine and DOPAC were electrochemically detected using a potentiostat (Antec Leyden, model Intro) fitted with a glassy carbon electrode set a +500 mV versus Ag/AgCl (Antec, Leyden). Data were analyzed using Chromatography Data System software (Shimadzu, Class-VP, Japan) using an external standard method to quantify concentrations of dopamine and DOPAC.

For statistical evaluation of the data, four consecutive predrug microdialysis samples with less than 50 % variation were used as the baseline and the mean of these samples was set to 100 %. Drug effects were expressed as percentages of basal level (mean ± SEM) within the same subject. Statistical analysis was performed using Sigmastat for Windows (SPSS Corporation). Drug effects were compared with baseline and vehicle control using a two-way ANOVA for repeated measurements followed by the Newman–Keuls post hoc test. The effect of haloperidol administration was compared to the vehicle control using a one-way ANOVA for repeated measurements followed by the Newman–Keuls post hoc test. For all tests, the level of statistical significance was set at *p* < 0.05.

### Regional analysis of GSK-3β phosphorylation state

The phosphorylation state of the protein kinase GSK-3β was measured in mice treated in vivo with doses of the typical antipsychotic drug, haloperidol, the atypical antipsychotic drug, clozapine, and ITI-007 that were selected based on those used in measurements of TH phosphorylation, as described above. The specificity of GSK-3β phosphorylation changes with regard to antipsychotic medications was evaluated by comparison with the antidepressant drug, imipramine (20 mg/kg, i.p.). Groups of mice (*N* = 4–6/treatment group) were treated with haloperidol (1 mg/kg, p.o.), clozapine (5 mg/kg, p.o.), ITI-007 (3 mg/kg, p.o.), or vehicle then killed at specified time points (120 min) postinjection by focused microwave cranial irradiation. Each brain was rapidly removed from the skull. Nucleus accumbens, striatum, and prefrontal cortex were dissected free-hand, frozen in liquid nitrogen, and stored at −80 °C until analyzed for phosphoprotein levels. Brain tissue was prepared for SDS-PAGE, and protein homogenates (15 μg) were separated on 10 % Bis-Tris gels, and proteins transferred to nitrocellulose membranes that were detected for phospho-serine (S) 9 GSK-3β (rabbit polyclonal) or total GSK-3β (rabbit polyclonal) (Cell Signaling, Danvers, MA). Antibody binding was revealed using fluorescent secondary antibodies and LiCor Odyssey software, as described above. Levels of phospho-GSK-3β were normalized for total levels of GSK-3β and expressed as a percent of phosphoprotein levels in the vehicle-injected controls.

### Analysis of GluN2B receptor phosphorylation state in the nucleus accumbens

The effect of ITI-007 on phosphorylation state of the GluN2B-type glutamate receptor was assessed in the nucleus accumbens. Mice (*N* = 8/treatment group) were treated with haloperidol (1 mg/kg, p.o.), ITI-007 (3 mg/kg, p.o.), or vehicle then killed 120 min postinjection by focused microwave cranial irradiation. Brains were rapidly removed from the skull. Nucleus accumbens was dissected free-hand from each mouse brain, frozen in liquid nitrogen, and stored at −80 °C until analyzed for phosphoprotein levels. Brain tissue was prepared for SDS-PAGE as described above, and protein homogenates (15 μg) were separated on 3–7 % gradient gels, and proteins transferred to nitrocellulose membranes. Portions of each membrane were immunoblotted for GluN2B phosphorylated at tyrosine (Y) 1472 (rabbit polyclonal) and total GluN2B (mouse monoclonal) (Thermo Fisher Scientific, Rockford, IL). Antibody binding was revealed using fluorescent secondary antibodies and LiCor Odyssey software, as described above. Levels of phospho-GluN2B were normalized for total levels of GluN2B and expressed as a percent of phosphoprotein levels in the vehicle-injected controls.

### Measurement of social interaction behavior in mice using the social defeat model

Mice were tested for social isolation behavior after repeated exposure (for 10 days) to an aggressive resident mouse in the social defeat paradigm as described by Berton et al. (2006). Mice (*N* = 8–12/treatment group) were then dosed chronically, once daily for 28 days, with either vehicle (5 % DMSO/5 % Tween 20/15 % PEG400/75 % water, 6.7 ml/kg volume) or ITI-007 (1 mg/kg, i.p.) in vehicle solution. On the day after the last drug or vehicle treatment, the mice were placed in an open field in the presence of a resident mouse (within a smaller cage) and the animal’s behavior recorded by videotape for 150 s. Videotracking software (Noldus, Netherlands) was used to calculate the total time each mouse spent in specified open-field quadrants, defined schematically in Fig. 8. The total time (s) spent by mice representing each drug treatment group in the interaction zone (Fig. 8) in proximity to the resident mouse or, in the corner zones, at a distance from the resident mouse (Fig. 8) was expressed as a mean (±SEM).

## Results

### Receptor binding profile analysis

**Fig. 1:**
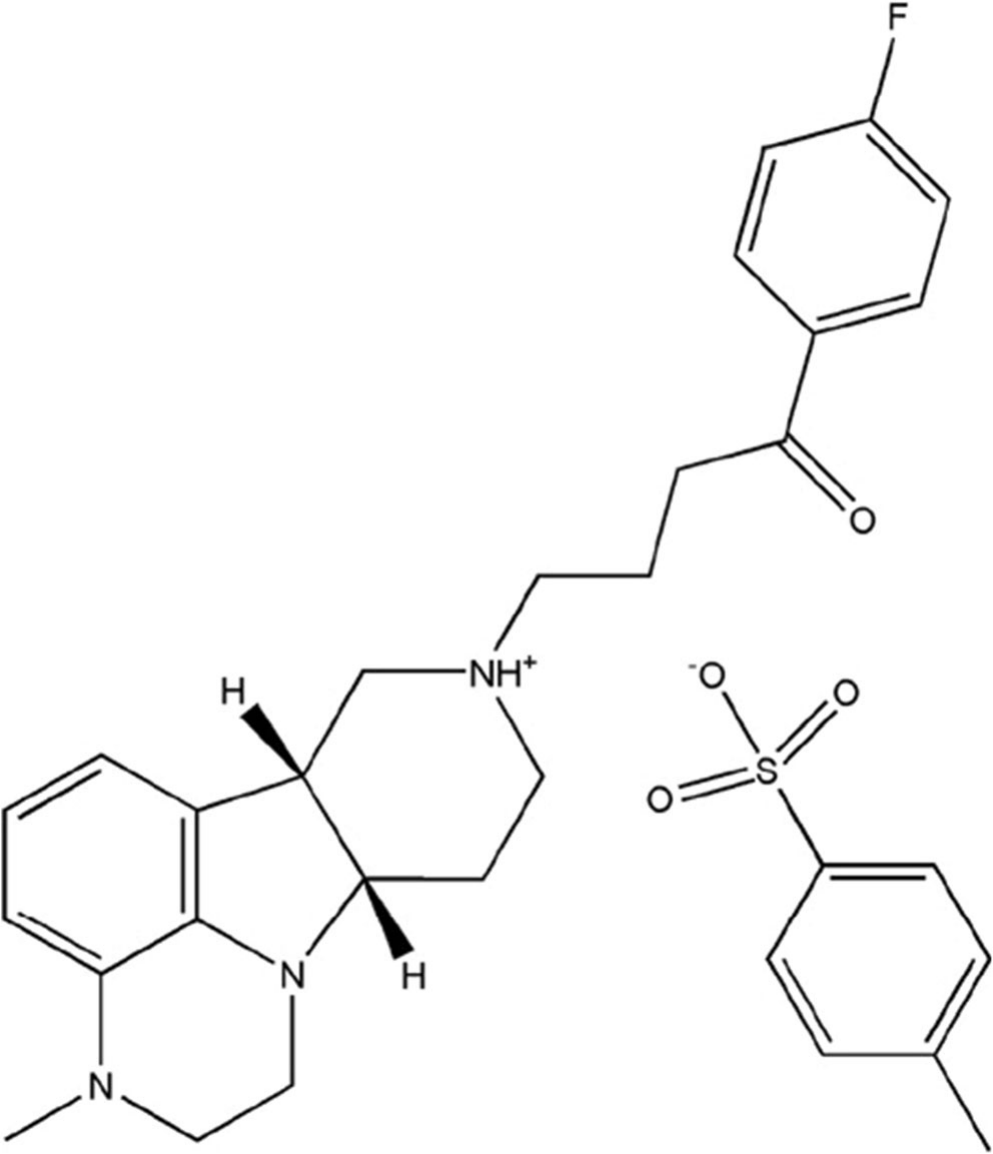
The structure of 1-(4-fluoro-phenyl)-4-((6bR,10aS)-3-methyl-2,3,6b,9,10,10a-hexahydro-1H,7H-pyrido[3′,4′:4,5]pyrrolo[1,2,3-de]quinoxalin-8-yl)-butan-1-one (ITI-007), the tosylate salt of IC200056

The structure of ITI-007, a tosylate salt, is shown in Fig. 1. Binding affinities of the compound to receptors implicated in the therapeutic actions of antipsychotic medications, including the serotonin 5-HT_2A_ receptors, dopamine D_2_ and D_1_ receptors, and the SERT are shown in Table 1. The binding affinities of several major atypical antipsychotic medications and the antidepressant medication, fluoxetine, derived from the NIMH Psychoactive Drug Screening Program (PDSP) Database (Roth et al. 2004), are presented for comparison in Table 1. ITI-007 displays high-affinity binding to the 5-HT_2A_ receptor with a *K*_*i*_ = 0.54 nM, and antagonized a serotonin-induced (30 nM) increase in calcium fluorescence (IC_50_ = 7 nM). The 5-HT_2A_ receptor is a G_q_-coupled receptor that functionally increases the turnover of phosphotidyl inositols in cells after stimulation with serotonin (Conn and Sanders-Bush 1984). In HEK293 cells, ITI-007 was found to antagonize serotonin-mediated increases in phosphatidylinositol (PI) turnover by right-shifting the serotonin dose–response curve, indicating activity as a 5-HT_2A_ antagonist. The compound also displays high-affinity binding toward dopamine D_2_ receptors, with a *K*_*i*_ = 32 nM (Table 1). Activation of D_2_ dopamine receptors inhibits adenylyl cyclase activity and results in a decrease in cyclic AMP accumulation (Stoof and Kebabian 1981). In a recombinant cell assay using CHO cells expressing a human recombinant D_2_-short receptor, ITI-007 functions as an antagonist at D_2_ dopamine receptors, blocking the ability of dopamine to inhibit forskolin-stimulated cAMP accumulation with an IC_50_ = 338 nM.

**Table 1:** Receptor binding affinity of ITI-007 as measured by radioligand displacement assays: comparison with antipsychotic and antidepressant medications

Receptor	ITI-007^a^	Risperidone^b^	Olanzapine^b^	Aripiprazole^b^	Fluoxetine^c^
5-HT_2A_	0.5	0.5	2.5	9	141
D_2_	32	5.9	31	1.6	>10,000
D_1_	52	564	128	1,170	>10,000
SERT	62	>1,000	>1,000	240–405	0.9–20
Ratios					
D_2_/5-HT_2A_	60	12	12.4	0.18	>70
Receptor					
H_1_ histamine	>1,000	14	2	28	1,240
5-HT_2C_	173	63	7.1	130	69
α_1_ Adrenergic	73	2.3	60	26	2,260
Ratios					
H_1_/5-HT_2A_	>2,000	28	1	3	8.8
5-HT_2C_/5-HT_2A_	320	126	3	14	0.5
α_1_/5-HT_2A_	135	5	24	2.9	16

^a^Binding affinities were determined as described in the “Materials and Methods” section;

^b^Binding affinities for other compounds were derived from the NIMH Psychoactive Drug Screening Program (PDSP) Database (Roth et al. 2004)

^c^Binding affinities for fluoxetine at 5-HT_2A_ (Owens et al. 1997), D_2_ (Sánchez and Hyttel 1999), D_1_ (Sánchez and Hyttel 1999), H_1_ (Owens et al. 1997, 2002), α_1_ adrenergic (Owens et al. 1997, 2002), and 5-HT_2C_ receptors (Pälvimäki et al. 1996; Bonhaus et al. 1997; Rothman et al. 2000; Sánchez and Hyttel 1999), as cited by from the NIMH Psychoactive Drug Screening Program (PDSP) Database

In order to profile side effect liabilities, binding affinity was determined at two receptors that are implicated in side effect liabilities for current antipsychotic medications, namely, the histamine H_1_ receptor and the serotonin 5-HT_2C_ receptor. The relative affinity of ITI-007 for these receptors, compared with exemplified antipsychotic medications, is shown in Table 1. ITI-007 possesses a high selectivity (i.e., >2,000-fold) for 5-HT_2A_ receptors relative to histamine H_1_ receptors. The compound also displays a high selectivity for 5-HT_2A_, relative to 5-HT_2C_ receptors (~320-fold 5-HT_2A_/5-HT_2C_ binding ratio) (Table 1).

### In vitro binding in a receptor selectivity panel

We evaluated potential off-target interactions using a broad panel of receptors and ion channels. The compound was screened at a single concentration of 100 nM across a custom panel of 66 receptors, ion channels, and enzymes, including receptors (i.e., H_1_ histaminergic and M_1_-M_5_ muscarinic subclasses) known to mediate certain adverse metabolic and cognitive side effects of antipsychotic medications. In this screen, ITI-007 displayed projected *K*_*i*_ values (>50 % binding inhibition) at or below 100 nM only at the 5-HT_2A_, D_1_, D_2_, D_4_, alpha_1A_ and alpha_1B_ receptors, and at SERT, confirming the receptor binding results (Table 1). The compound did not bind significantly to any of the other targets studied (Table 2).

**Table 2:** Receptor binding selectivity of ITI-007 in vitro as measured against a broad specificity profile panel: off-target receptor interactions with >50 % binding at a 100 nM concentration of ITI-007 (of a total of 66 substrates evaluated)

Substrate	% Inhibition of binding at 100 nM concentration^a^
Adrenergic, 1β	85.43
Adrenergic, α_1_	66.06
Dopamine, D_4_	64.27

^a^Values are expressed as the percent inhibition of specific binding and represent the average of replicate tubes at the specified drug concentration

### Assessment of antipsychotic-like activity

**Fig. 2:**
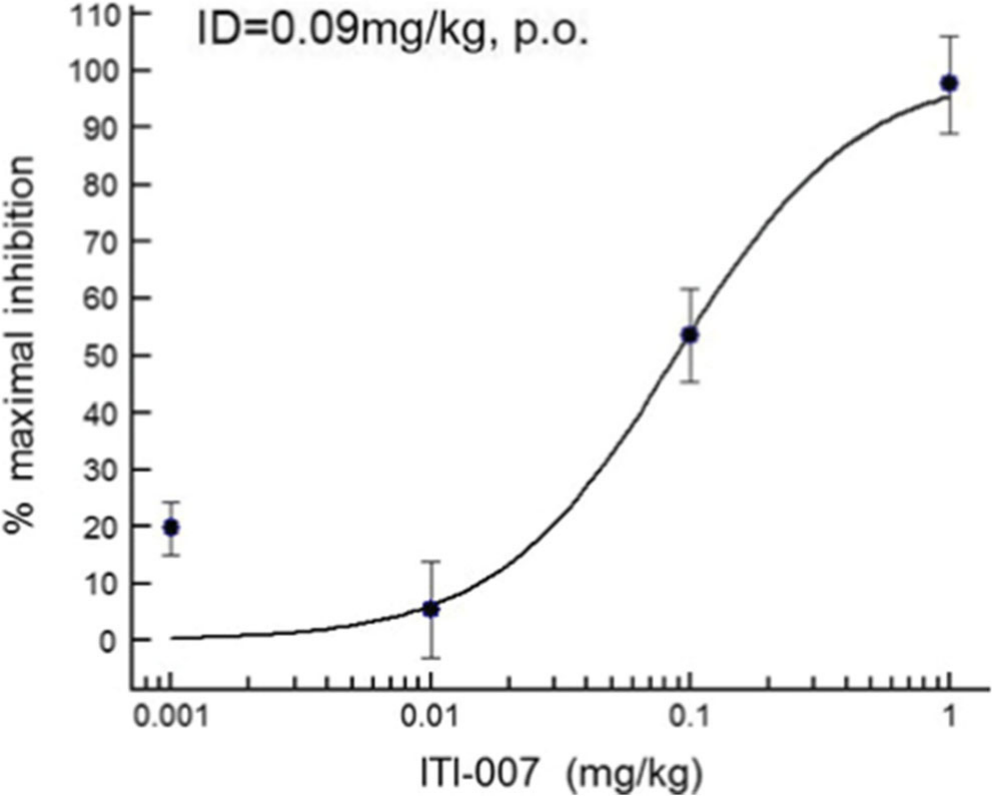
Dose–response curve for inhibition of DOI-induced headtwitch behavior by ITI-007 in mice. The 5-HT_2A_ agonist, DOI, was used to elicit stereotyped headtwitch behavior in mice. Mice (*N* = 4/group) were given a specified oral dose of ITI-007 (0.001–1 mg/kg in 0.5 % methylcellulose in water) or vehicle (0.5 % methylcellulose). Thirty minutes later, the mice were injected with vehicle (saline) or with the 5-HT_2A_ agonist, DOI (2.5 mg/kg, i.p., in saline). Headtwitches were then counted for 5 min, starting 10 min after DOI injection. The mean (±SEM) number of headtwitches recorded in vehicle-treated mice was 13.7 ± 0.67. An ID_50_ for inhibition of DOI-induced headtwitch was calculated using a four-parameter logistical fit (Excel Fit software, IDBS)

Functional activity as a 5-HT_2A_ receptor antagonist in vivo was measured by blockade of 5-HT_2A_ agonist (DOI) headtwitch behavior in mice. When administered orally to mice, the compound effectively blocked the appearance of headtwitch behaviors induced by DOI (2.5 mg/kg, i.p.) (Fig. 2) with a calculated ID_50_ of 0.09 mg/kg (p.o.). These data are consistent with a good oral exposure and functional activity of ITI-007 in vivo as a 5-HT_2A_ receptor antagonist.

**Fig. 3:**
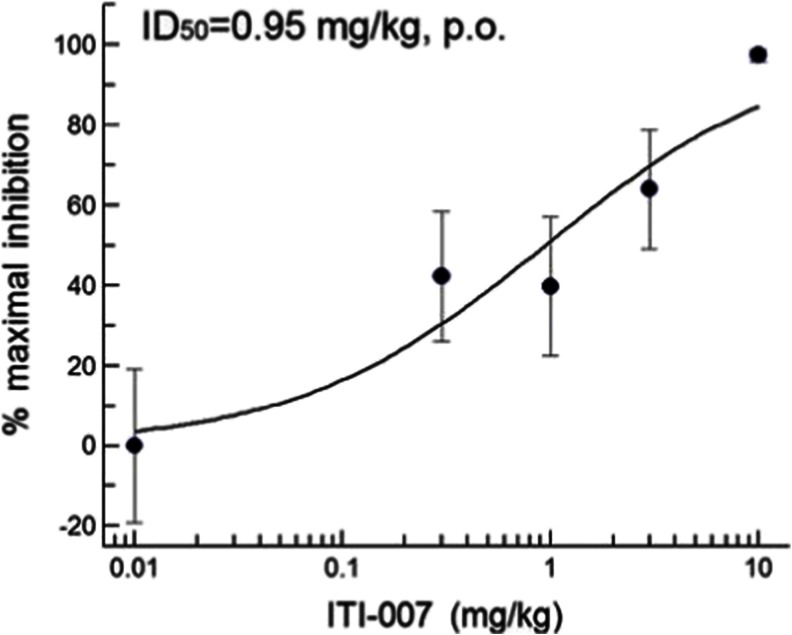
Dose–response curve for inhibition of AMPH-induced hyperlocomotion by ITI-007 in rats. The psychostimulant drug D-amphetamine was used to elicit hyperlocomotion in rats. Sprague–Dawley rats (*N* = 4/group) were habituated to locomotor activity chambers (AccuScan, Columbus, OH) for 60 min then given a specified oral dose of ITI-007 (0.3–10 mg/kg, in 0.5 % methylcellulose in water, p.o.) or vehicle (0.5 % methylcellulose in water). Thirty minutes later, the rats were injected with vehicle (saline, i.p.) or with D-amphetamine (D-AMPH) (1 mg/kg, in saline, i.p.) and locomotor activity monitored for an additional 2 h. Total distance traveled was quantitated and averaged for each treatment group. The mean (+SEM) total activity (centimeters traveled) recorded for vehicle-treated rats given D-AMPH was 21,583 ± 4,153. Percent inhibition of each ITI-007 treatment group compared with D-AMPH group was calculated. The activity level in the D-AMPH + vehicle group was used to determine 0 % inhibition. Data were analyzed to determine an ID_50_ using a four-parameter logistical fit (Excel Fit software, IDBS)

Postsynaptic dopamine D_2_ antagonist activity was tested in vivo using the rat D-AMPH hyperactivity assay. Rats were pretreated with specified oral doses of ITI-007 (0.3–10 mg/kg, p.o.) or vehicle 30 min prior to an i.p. injection of the psychostimulant D-AMPH (1 mg/kg). Locomotor activity was recorded, and the ID_50_ value was then calculated. The compound blocked hyperactivity with an ID_50_ = 0.95 mg/kg (p.o.) (Fig. 3). This value is similar to that determined for the antipsychotic medication, risperidone, which displayed an ID_50_ = 0.33 mg/kg (p.o.) in this assay. Two antipsychotic medications, the typical antipsychotic haloperidol, and the atypical antipsychotic, aripiprazole, were also tested. As anticipated, haloperidol, a potent neuroleptic, blocked psychostimulant-induced hyperactivity with an ID_50_ = 0.04 mg/kg (p.o.). Aripiprazole, a medication reported to display mixed D_2_ agonist/antagonist activity (Burris et al. 2002), was less potent, blocking hyperlocomotion with an ID_50_ = 4.65 mg/kg (p.o.). The ID_50_ values calculated here for risperidone, haloperidol, and aripiprazole are consistent with published potencies reported for these compounds (Gyertyán et al. 2011).

### Effect on phosphorylation of TH

**Fig. 4:**
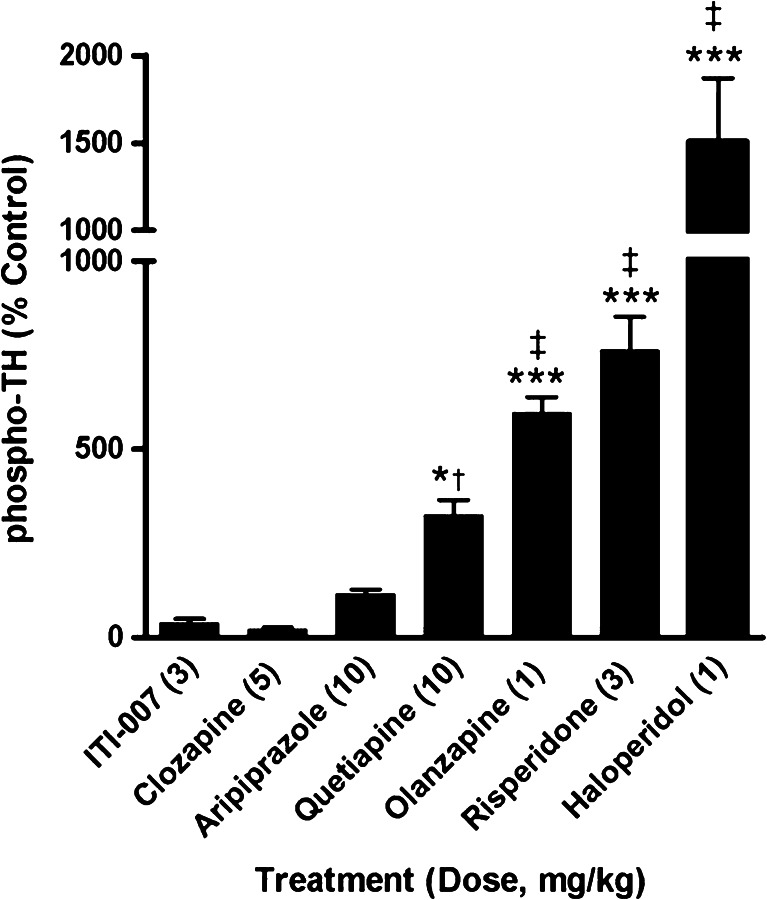
Comparison of the effect of antipsychotic medications with ITI-007 on the phosphorylation state of striatal TH in vivo. Mice (*N* = 6/treatment group) were treated acutely with behaviorally efficacious doses of ITI-007 (3 mg/kg, p.o.), clozapine (5 mg/kg, i.p.), aripiprazole (10 mg/kg, p.o.), quetiapine (10 mg/kg, i.p.), olanzapine (1 mg/kg, i.p.), risperidone (3 mg/kg, p.o.), or haloperidol (1 mg/kg, i.p.) then killed 15, 30, or 60 min later. The change in phosphorylation state at serine (S) 40 of tyrosine hydroxylase (TH) was determined in striatal samples by Western blotting using a phosphorylation-state specific S40 antibody. Phosphoprotein levels were normalized for the total level of phosphoprotein in the sample as detected by a pan-TH antibody. Integrated changes in phosphorylation state were calculated, relative to control samples, over the 60-min period after drug treatment for each compound. *p < 0.01; ***p < 0.001 compared with control, ^‡^ *p* < 0.001 compared with ITI-007, clozapine, and aripiprazole; ^†^ *p* < 0.05 compared with aripiprazole, ANOVA with Newman–Keuls post hoc test

We monitored phosphorylation of tyrosine hydroxylase (TH), which is localized in presynaptic terminals of dopamine neurons in striatum, as a functional indicator for the potential of antipsychotic drugs to disrupt striatal dopamine metabolism. Phosphorylation of the enzyme at serine 40 (S40) is essential for catalytic activity; increased S40 phosphorylation results in increased dopamine biosynthesis in response to blockade of D_2_ autoreceptors on these terminals (Harada et al. 1996). Drug effects on S40 phosphorylation were analyzed for a panel of psychoactive drugs and for ITI-007 using CNSProfile™, an immunoblotting platform developed at Intra-Cellular Therapies, Inc. Dose levels of drugs were chosen for comparative analysis based on reported efficacious dose ranges of each drug in rodent tests of antipsychotic activity, including the conditioned avoidance response and amphetamine hyperactivity paradigms (Wadenberg et al. 2001; Brennan et al. 2010). CNSProfile™ analysis revealed a robust increase in phosphorylation state of the S40 residue on TH for most typical and atypical antipsychotic drugs studied compared with vehicle controls. The typical antipsychotics, haloperidol, and atypical antipsychotics which possess high-affinity D_2_ receptor antagonist activity, such as risperidone and olanzapine, were found to significantly increase TH phosphorylation (Fig. 4). In contrast, drugs with partial D_2_ agonist activity, such as aripiprazole, and medications with mixed dopamine receptor activities, such as clozapine (Factor and Friedman 1997), did not significantly affect TH phosphorylation state. ITI-007, administered at a dose level (3 mg/kg, p.o.) above the IC_50_ for blockade of D-AMPH hyperactivity (~1.0 mg/kg, p.o.) had no significant effect on phosphorylation at S40 (Fig. 4), showing a biochemical response at this site similar to that of aripiprazole and clozapine.

### Effect on dopamine neurotransmission

**Fig. 5:**
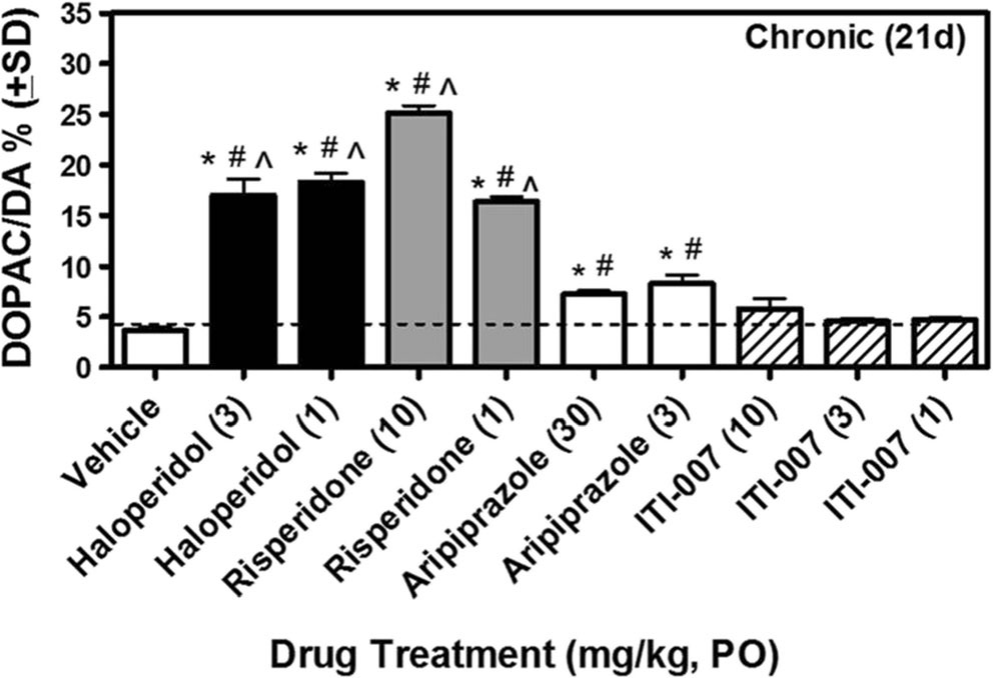
Effect of chronic (21 day) daily administration of haloperidol, risperidone, aripiprazole, or ITI-007 on striatal dopamine metabolism in vivo. Mice (*N* = 6/dosing group) received an oral dose of vehicle (5 % gum arabic in water, 6.7 ml/kg volume, p.o.) or vehicle solution containing either haloperidol (1 or 3 mg/kg), risperidone (1 or 10 mg/kg), aripiprazole (3 or 30 mg/kg), or ITI-007 (1, 3, or 10 mg/kg) once daily for 21 days. Animals were killed by focused cranial microwave irradiation 2 h after the last drug dose. Striatum was collected for analysis of levels of dopamine and dopamine metabolites, DOPAC and HVA, using HPLC-EC. DOPAC/DA ratio, used as an index of dopamine synthetic rate, is shown. *p < 0.05 compared with vehicle alone; ^#^ *p* < 0.05 compared with ITI-007 (3); ^^^ *p* < 0.05 compared with ITI-007 (10)

To directly measure effects of ITI-007 on striatal dopamine neurotransmission, we monitored the impact of this drug on striatal dopamine turnover in mice at dose levels comparable to those studied for TH phosphorylation. Mice were given haloperidol (1 or 3 mg/kg), risperidone (1 or 10 mg/kg), aripiprazole (3 or 30 mg/kg), ITI-007 (1, 3, or 10 mg/kg), or vehicle once (acutely) or once daily for 21 days (chronically) and then killed 2 h after the last drug dose. Striatal dopamine metabolism was monitored by measurement of levels of dopamine (DA), DOPAC, and HVA. Acute or chronic administration of haloperidol or risperidone resulted in significant increases in the metabolism of dopamine, as measured by elevated DOPAC/DA and HVA/DA ratios (Fig. 5; Table 3). Aripiprazole, which has been previously reported to have a low liability for alteration of striatal dopamine metabolism (Nakai et al. 2003) owing to partial agonist properties at presynaptic D_2_ receptors, had a small effect on DOPAC/DA and HVA/DA ratios that was statistically significant after acute and chronic drug administration compared with vehicle control (Table 3; Fig. 5). ITI-007, administered at three dose levels representing a ~10-fold range encompassing the effective dose level for blockade of D-AMPH hyperactivity (IC_50_ = 0.95 mg/kg, p.o.), had no significant effect on the DOPAC/DA or HVA/DA ratio, relative to vehicle control, measured after either acute or chronic administration (Fig. 5; Table 3).

**Table 3:** Effect of acute (2 h) or chronic (21 day) daily treatment with haloperidol, risperidone, aripiprazole, or ITI-007 on HVA/DA and DOPAC/DA ratios in rat striatal tissue

Compound (dose in mg/kg)	Acute (2 h) dosing				Chronic (21 day) dosing			
	HVA/DA		DOPAC/DA		HVA/DA		DOPAC/DA	
	Mean	SD	Mean	SD	Mean	SD	Mean	SD
Vehicle (0)	6.59	0.62	4.58	0.51	7.04	0.41	3.61	0.81
Haloperidol (3)	28.14*,**,***	2.08	22.68*,**,***	2.80	24.15*,**,***	4.89	17.00*,**,***	3.94
Haloperidol (1)	31.04*,**,***	1.84	19.69*,**,***	1.45	29.23*,**,***	4.85	18.26*,**,***	2.21
Risperidone (10)	23.06*,**,***	3.80	30.98*,**,***	2.68	36.39*,**,***	0.54	25.12*,**,***	1.91
Risperidone (1)	30.71*,**,***	1.86	14.35*,**,***	2.46	29.37*,**,***	2.26	16.35*,**,***	1.25
Aripiprazole (30)	11.20	0.90	11.89	0.96	12.32*	1.30	7.25*,**	0.80
Aripiprazole (3)	12.63	1.39	8.57	0.96	12.14*	1.71	8.27*,**	2.08
ITI-007 (10)	8.40	0.63	6.02	0.59	10.47	2.89	5.80	2.45
ITI-007 (3)	7.47	0.98	4.57	0.48	7.99	1.24	4.54	0.73
ITI-007 (1)	8.06	1.37	4.98	0.80	9.44	4.34	4.69	0.61

*p < 0.05 compared with vehicle alone; **p < 0.05 compared with ITI-007 (3); ***p < 0.05 compared with ITI-007 (10)

### Measurement of forelimb catalepsy in mice

**Fig. 6:**
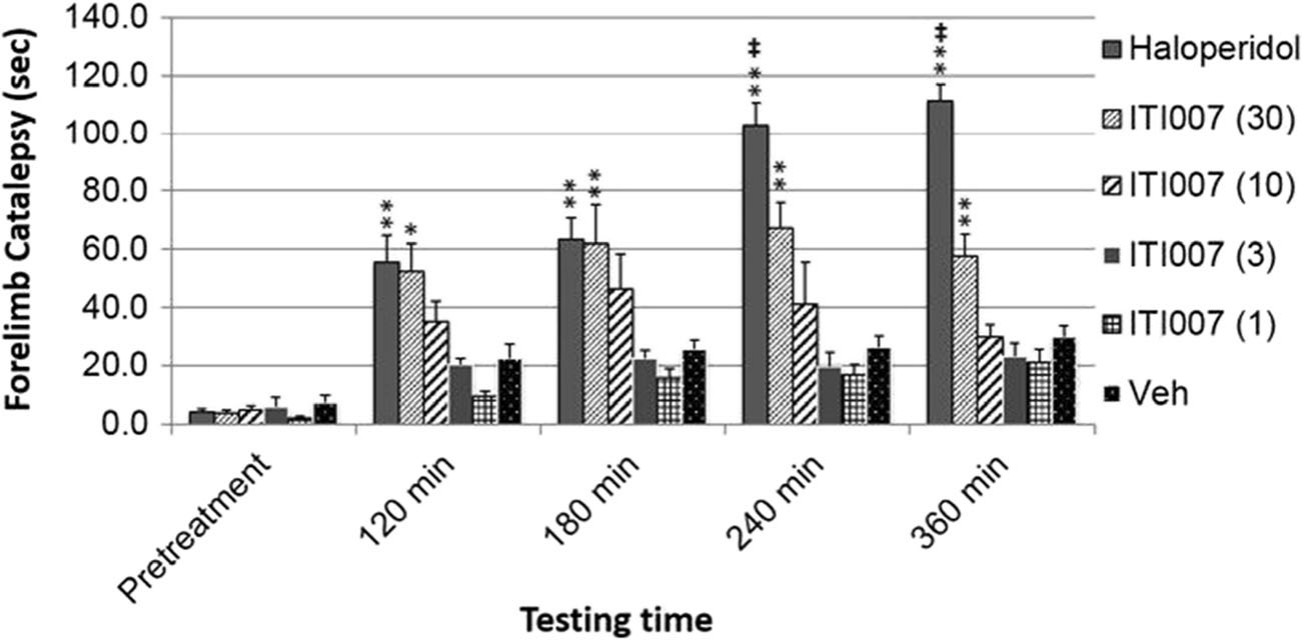
Effect of haloperidol and ITI-007 on motor performance as measured by forelimb catalepsy. Forelimb catalepsy was measured in mice using the bar grip test. Animals received a single oral dose of vehicle (Veh) (0.5 % methylcellulose in water, 6.7 ml/kg volume, p.o.) or haloperidol (3 mg/kg) or ITI-007 (1–30 mg/kg) in vehicle solution. Catalepsy was then measured in mice (*N* = 4/dose/drug) by recording the latency (in seconds) to step both front paws down to the floor of the cage up to a maximum time of 120 s. Catalepsy scores were recorded for each mouse at 120, 180, 240, and 360 min after drug administration. Mean forelimb catalepsy time (in seconds) was calculated across each group and time point. Data were analyzed using ANOVA with Newman–Keuls post hoc test. Data are presented as mean ± SEM. *p < 0.05; **p < 0.01 compared with vehicle treatment. ^‡^ *p* < 0.01, statistically significant difference between haloperidol and ITI-007 treatments

To further examine the potential for motor side effects by ITI-007, we tested the compound for induction of forelimb catalepsy in mice. Mice administered an oral dose of the compound displayed a statistically significant increase in forelimb catalepsy, as measured in the bar grip test, only at the highest dose level tested, 30 mg/kg. The effect never reached maximal cutoff times (i.e., 120 s), suggesting a lack of frank catalepsy (Fig. 6). Mice receiving dose levels of 1–10 mg/kg did not exhibit significant forelimb catalepsy, compared with vehicle-injected control mice. In contrast, mice receiving oral administration of haloperidol exhibited profound forelimb catalepsy at each time point measured, compared with vehicle-injected mice. Further, haloperidol-treated mice displayed significantly higher latencies to move off of the bar, compared with mice treated with a 30 mg/kg dose of ITI-007 at 240- and 360-min time points (Fig. 6).

### Dopamine release in prefrontal cortex

**Fig. 7:**
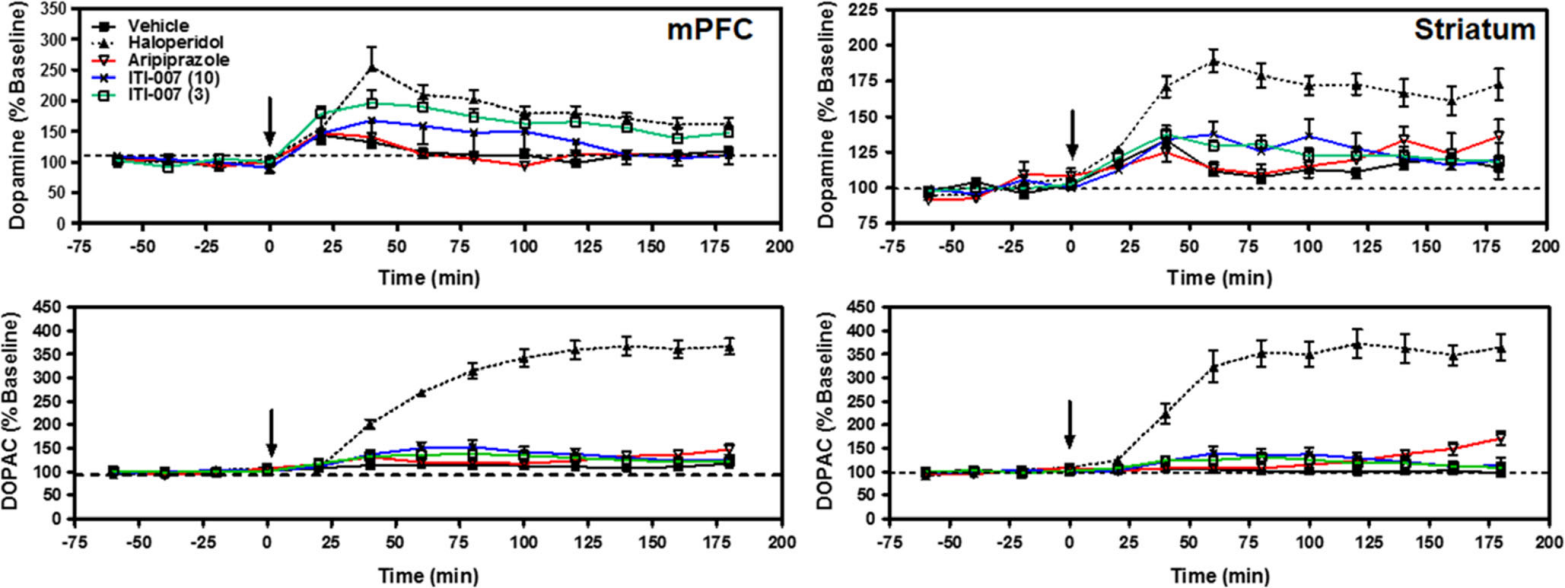
Effect of acute administration of haloperidol, aripiprazole, or ITI-007 on extracellular dopamine and DOPAC levels in rat striatum and medial prefrontal cortex, as measured by in vivo microdialysis. Adult, male Wistar rats were surgically prepared with microdialysis probes for collection of dialysate from both medial prefrontal cortex (mPFC) and striatum. Following establishment of baseline DA and DOPAC levels, the rats received (at *t* = 0 min, designated by *arrow*) an acute dose of vehicle solution (0.5 % methylcellulose in water, 1 ml/kg volume, p.o.; *N* = 8–9 rats; *filled box*), haloperidol (0.3 mg/kg in acidified water, 1 ml/kg, s.c.; *N* = 6–10 rats; *filled triangle*), aripiprazole (30 mg/kg, p.o.; *N* = 5–6 rats; *open red triangle*), or ITI-007 (3 or 10 mg/kg, p.o.; *N* = 6–10 rats each; *open green box* and *cross*, respectively). Striatal and mPFC dialysates were collected every 20 min for 3 h for measurement of dopamine (*top panels*) and DOPAC (*bottom panels*). Analysis of variance with Newman–Keuls post hoc tests revealed significant effects, compared with vehicle control, of haloperidol on DA efflux in mPFC (*p* < 0.01) and striatum (*p* < 0.001) and DOPAC efflux in mPFC and striatum (*p* < 0.001). ITI-007 (3 mg/kg) induced a significant increase in DA efflux, compared to vehicle control, in mPFC (*p* < 0.05). The increase in DA efflux in mPFC induced by ITI-007 (3 mg/kg) was significantly larger than that induced by aripiprazole (30 mg/kg) (*p* < 0.05)

Since the selectivity of certain antipsychotic medications for dopamine effects in mesocortical/mesolimbic systems compared with nigrostriatal systems has been hypothesized as a critical factor in the lower liability of these compounds for motor side effects, including tardive dyskinesia and extrapyramidal motor symptoms (Moghaddam and Bunney 1990; Ichikawa et al. 2002; Svensson et al. 1993; Kane et al. 2002), we measured the ability of ITI-007 to increase extracellular levels of dopamine in the medial prefrontal cortex (mPFC), compared with the striatum using in vivo microdialysis. The typical antipsychotic drug, haloperidol, and the atypical antipsychotic drug, aripiprazole, were tested in parallel. Dopamine and DOPAC levels were measured simultaneously in rats prepared with microdialysis probes in both the mPFC and striatum (*N* = 6–10/group). Vehicle injection resulted in no significant change in either DA or DOPAC levels in the dialysate samples from either striatum or mPFC (Fig. 7). As anticipated, administration of haloperidol (0.3 mg/kg) resulted in a significant increase in both DA and DOPAC efflux in rat striatum (*p* < 0.001 for DA and DOPAC levels, compared with vehicle, ANOVA, Newman–Keuls post hoc test). DA and DOPAC levels in mPFC were also elevated after haloperidol treatment (*p* < 0.01 and *p* < 0.001 for DA and DOPAC levels, respectively, compared with vehicle, ANOVA, Newman–Keuls posttest). ITI-007 treatment (3 or 10 mg/kg) induced dose-dependent effects on DA efflux only in mPFC (Fig. 7). ITI-007, significantly increased DA (but not DOPAC) efflux in mPFC at a 3 mg/kg dose (*p* < 0.05 for DA levels compared with baseline, ANOVA, Newman–Keuls posttest). At a higher dose level (10 mg/kg), the compound was associated with a trend toward an increase in DA efflux in mPFC that was not statistically significant. ITI-007, at a 3 mg/kg dose, resulted in a significantly larger increase in mPFC DA efflux than a 30 mg/kg dose of aripiprazole (*p* < 0.05). Aripiprazole administration did not significantly affect either DA or DOPAC efflux in rat striatum or mPFC at the dose tested (30 mg/kg). In summary, ITI-007 preferentially increased DA efflux in the mPFC compared with striatum. Moreover, at the doses tested, ITI-007 treatment induced a significantly larger increase in cortical DA efflux, relative to vehicle, than aripiprazole did (Fig. 7).

### Effects on GSK-3β phosphorylation state

Given the microdialysis data indicating preferential effects of ITI-007 on prefrontal cortex dopamine neurotransmission (a target of the mesocortical dopamine pathway) compared with the striatum, (a target of the nigrostriatal dopamine pathway) we used CNSProfile to identify other biochemical signatures of this effect.

Treatment of mice with a typical (haloperidol) or an atypical (clozapine) antipsychotic medication resulted in distinct regional effects on GSK-3β phosphorylation at S9 (Table 4). The administration of clozapine (5 mg/kg, p.o.) led to a significant increase in phospho-S9 GSK-3β in both prefrontal cortex (140 ± 5.8 % of control, *p* < 0.01) and nucleus accumbens (125 ± 10 % of control, *p* < 0.01) but did not significantly affect striatal GSK-3β phosphorylation (117 ± 23.5 % of control, *p* > 0.05). In contrast, haloperidol treatment (1 mg/kg, p.o.) had no significant impact on phospho-S9 levels in prefrontal cortex (96 ± 2.6 % of control, *p* > 0.05) or nucleus accumbens (99.4 ± 4.2 % of control; *p* > 0.05) (Table 4). A trend toward an increase in GSK-3β phosphorylation was noted with haloperidol in the striatum but failed to reach statistical significance (114.7 ± 3.5 %, *p* > 0.05). ITI-007 administration (3, 10, or 30 mg/kg, p.o.) induced a dose-dependent increase in GSK-3β phosphorylation at S9 in prefrontal cortex and nucleus accumbens with maximal effects seen at 10 mg/kg (p.o.) in both brain regions (126 ± 4.9 % in prefrontal cortex, *p* < 0.001; 120.8 ± 3.6 % in nucleus accumbens, *p* < 0.001). ITI-007 had no significant effect on S9 phosphorylation in striatum at any dose tested (Table 4). Treatment with imipramine, an antidepressant medication, had no significant effect on GSK-3β phosphorylation state in any of the three brain regions evaluated.

**Table 4:** Regional effects of ITI-007, clozapine, haloperidol, and imipramine on GSK-3β phosphorylation state (mean ± SEM) in mouse brain

Phospho-S9 GSK-3β (% vehicle control)							
Dose (mg/kg)	ITI-007				Clozapine	Haloperidol	Imipramine
	0	3	10	30	5	1	20
Accumbens	100 ± 3.0	122.9 ± 8.6**	120.8 ± 3.6***	105.5 ± 2.2	125.9 ± 10** (100 ± 2.8)^a^	99.4 ± 4.2 (100 ± 3.3)^a^	108.7 ± 6.2 (100 ± 1.9)^a^
PFC	100 ± 2.1	103.4 ± 3.1	126.0 ± 4.9***	112.1 ± 2.7*	140 ± 5.8** (100 ± 4.0)^a^	96.2 + 2.6 (100 ± 4.0)^a^	92 ± 4.6 (100 ± 5.9)^a^
Striatum	100 ± 2.9	106.6 ± 2.1	98.2 ± 5.1	108.0 ± 2.9	117.5 ± 23.5 (100 ± 3.2)^a^	114.7 ± 3.5 (100 ± 3.3)^a^	106.4 ± 8.6 (100 ± 3.7)^a^

*p < 0.05; **p < 0.01; ***p < 0.001 compared with vehicle (0) ANOVA with Newman–Keuls post hoc test

^a^Control values ± SEM

### Effects on GluN2B receptor phosphorylation state

We investigated the impact of haloperidol and ITI-007 on regulation of GluN2B receptors at Y1472 in nucleus accumbens. Haloperidol treatment (1 mg/kg, p.o.) increased GluN2B phosphorylation at Y1472 in the nucleus accumbens measured 120 min after drug administration (177 ± 28 % of control). ITI-007 (3 mg/kg, p.o.) also significantly increased phospho-Y1472 levels in mouse nucleus accumbens with maximal effects measured 120 min after dosing (180 ± 20 % of control). The data support the concept that ITI-007 exerts molecular effects in the nucleus accumbens that promote glutamatergic neurotransmission.

### Social interaction behavior following repeated social defeat

**Fig. 8:**
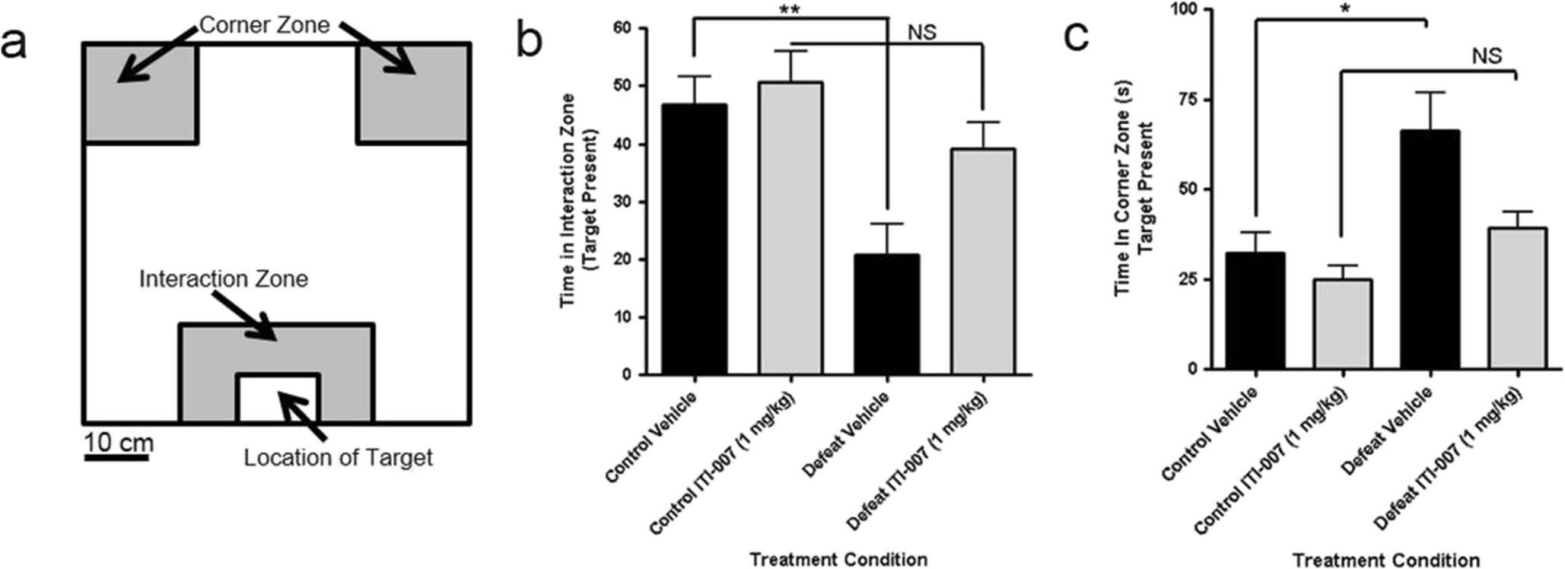
Effect of chronic administration of ITI-007 on social behavior following repeated social defeat. Mice (*N* = 8–12/treatment group) were subjected to exposure to an aggressive resident mouse in the social defeat/resident intruder paradigm. They were then dosed once daily for 28 days, with either vehicle (5 % DMSO/5 % Tween 20/15 % PEG400/75 % water, 6.7 ml/kg volume) or ITI-007 (1 mg/kg, ip.) in vehicle solution. On the day after the last drug or vehicle treatment, mice were placed in the open field in the presence of a resident mouse (enclosed in a smaller cage) and the animal’s behavior recorded by videotape for 150 s. Videotracking software was employed to calculate the time spent by each mouse in specified open-field quadrants, defined schematically in **a**. The total time (s) spent by each drug treatment group in the interaction zone (**b**) in proximity to the resident mouse or in the corner zones, at a distance from the resident mouse (**c**) was expressed as a mean (±SEM). *p < 0.05; **p < 0.01 compared with control vehicle; *NS* not significantly different from drug-treated control

We used the social defeat model to assess the ability of ITI-007 to attenuate reductions in socialization following chronic stress. Antidepressant medications with potent SERT activity, including fluoxetine, reverse stress-induced social withdrawal in this paradigm (Berton et al. 2006; Krishnan and Nestler 2011). Mice were exposed to an aggressive resident mouse for 10 min daily for 10 days then dosed chronically, once daily for 28 days, with either vehicle or ITI-007 (1 mg/kg, i.p.) in vehicle. On the day after the last drug or vehicle treatment, mice were placed in the open field in the presence of a resident mouse (enclosed in a smaller cage) and the total time each test mouse spent during a 150 s period in defined open-field quadrants in close proximity to another aggressive resident (i.e., interaction zone, Fig. 8b) or in isolation from the resident (i.e., the corner zones, Fig. 8c) was measured. As anticipated, chronic social defeat significantly reduced the amount of time test mice spent in proximity to the social target (*p* < 0.0.05 compared with vehicle). However, mice treated with ITI-007 following exposure to the defeat paradigm, showed no such reduction in social behavior (not significant compared with ITI-007 alone). Treatment with the compound alone did not result in differences in time spent in the interaction zone, compared with untreated control mice.

## Discussion

These experiments characterize ITI-007 as a novel small-molecule therapeutic agent displaying the combined properties of potent 5-HT_2A_ antagonism, cell type-specific modulation of dopamine protein phosphorylation pathways, and SERT binding with activity in preclinical screens predicting antipsychotic and antidepressant efficacy.

### Receptor binding profile

Radioligand displacement studies and in vitro functional activity assays confirmed a potent (*K*_*i*_ = 0.54 nM) binding affinity of ITI-007 for the 5-HT_2A_ subclass of serotonin receptors, expressed as antagonism of 5-HT-induced inositol phosphate signaling, comparable to current antipsychotic medications such as risperidone (*K*_*i*_ = 2 nM). ITI-007 did not significantly bind 5-HT_2B_ receptors, which mediate the adverse mitral valve damage in the cardiovascular system previously associated with weight loss drug “fen-phen” (Rothman et al. 2000; Fitzgerald et al. 2000). The compound also showed no significant binding to histamine H_1_ receptors or muscarinic cholinergic receptors (e.g., M_3_ subclasses), implicated in mediating the sedation associated with antipsychotic medications (Nasrallah 2008; Patel et al. 2009; Reynolds and Kirk 2010). The compound did not significantly bind 5-HT_2C_ receptors. Both 5-HT_2C_ receptors and H_1_ receptors have been implicated in the adverse effects of atypical antipsychotic medications on lipid metabolism and weight gain (Lieberman et al. 2005; Nasrallah 2008; Patel et al. 2009; Reynolds and Kirk 2010). The compound showed binding to alpha_1A_ and _1B_ adrenergic receptors with an affinity (*K*_*i*_ = 173 nM for α_1_) far below that of the 5-HT_2A_ receptor. A broad specificity panel survey of 66 receptor targets failed to identify other significant target interactions. In summary, ITI-007 displayed low affinity for targets associated with major liabilities of current antipsychotic medications, including metabolic disturbances, weight gain, and orthostatic hypotension.

### Separation of 5-HT_2A_ and dopaminergic activities

A key feature of this investigational compound is the 60-fold separation between its affinity for 5-HT_2A_ receptors and D_2_ receptors (*K*_*i*_ = 32 nM) or the SERT (*K*_*i*_ = 62 nM). This property predicts that lower concentrations of the drug may have behavioral effects predominantly mediated by 5-HT_2A_ receptor antagonism and that as the concentration is increased, additional pharmacological effects will emerge. This separation of serotonergic and dopaminergic activities is unprecedented among the currently used antipsychotic medications, including risperidone (ratio of 12-fold), olanzapine (12.4-fold), and aripiprazole (ratio of 0.18-fold). Concomitant 5-HT_2A_ and D_2_ modulation limit the use of these compounds for indications that might benefit from more highly selective 5-HT_2A_ antagonism. For example, selective 5-HT_2A_ receptor antagonists promote slow wave sleep and improve sleep consolidation (Ancoli-Israel et al. 2011; Morairty et al. 2008; Popa et al. 2005; Vanover and Davis 2010). Importantly, adding 5-HT_2A_ receptor antagonism enhances antipsychotic-like efficacy and reduces side effects of relatively selective D_2_ receptor antagonists, such as haloperidol, and of mixed 5-HT_2A_ and D_2_ receptor antagonists that do not already have a high separation of 5-HT_2A_ and D_2_ receptor antagonism, such as risperidone (Gardell et al. 2007). The high potency of ITI-007 for 5-HT_2A_ receptors (subnanomolar) coupled with the ~60-fold separation of 5-HT_2A_ to D_2_ receptor affinities is a likely explanation for the minimal catalepsy observed here in mice treated with the drug (Ohno et al. 1994).

### Minimal perturbation of striatal dopamine neurotransmission

The dopamine D_2_ receptor binding affinity of ITI-007 was comparable with current antipsychotic medications, including olanzapine (*K*_*i*_ = 31 nM) and risperidone (*K*_*i*_ = 5.9 nM) (NIMH PDSP Database) (Roth et al. 2004). D-AMPH-induced hyperlocomotion was blocked in rats by ITI-00*7*, consistent with a reduction in dopamine D_2_ receptor stimulation, and increased dopamine efflux in the mPFC, consistent with potent antagonism at D_2_ receptors. Although other aspects of its mechanism of action, such as 5-HT_2A_ receptor antagonism, may indirectly contribute to these pharmacological effects (Auclair et al. 2004; Ichikawa et al. 2001), the absence of functional effects on multiple measures of striatal dopamine neurotransmission indicate that ITI-007 also may act as a partial agonist at presynaptic dopamine D_2_ receptors. Most current antipsychotic agents, including haloperidol, risperidone, and olanzapine, significantly disrupt striatal dopamine neurotransmission, measured as increased dopamine turnover (Nakai et al. 2003), or as increased TH phosphorylation (Fig. 4), because they block presynaptic D_2_ autoreceptors (Nakai et al. 2003). In contrast, ITI-007 had no effect on presynaptic dopamine measures, including dopamine turnover (i.e., DOPAC/DA ratio), TH phosphorylation, or dopamine overflow, at dose levels that blocked D-AMPH hyperlocomotion. Furthermore, no striatal-based motor side effects (i.e., catalepsy) were observed in response to ITI-007. The collective profile in assays of in vivo striatal biochemistry and behavior for this drug is similar to that of other molecules with partial agonist activity at D_2_ receptors. For example, 3-(3-hydroxyphenyl)-N-*n*-propylpiperidine (3PPP), an early example of a D_2_ partial agonist (Clark et al. 1984), is thought to “stabilize” presynaptic D_2_ autoreceptors, thereby dampening perturbations in dopamine tone. Aripiprazole, an antipsychotic medication used as a comparator in the current study, demonstrated partial agonist activity at presynaptic D_2_ receptors that is believed responsible for its reduced motor side effects (Burris et al. 2002). Our data suggest that ITI-007 possesses functional activity as an antagonist at postsynaptic D_2_ receptors and a partial agonist at presynaptic striatal D_2_ receptors, which results in robust antipsychotic activity and a lack of motor side effects in relevant animal models.

### Preferential effects on mesocortical/mesolimbic pathways

ITI-007 preferentially modulated mesolimbic and mesocortical markers of dopamine neurotransmission. The compound dose-dependently increased phosphorylation of GSK-3β at a residue (serine 9) that inhibits kinase activity (Sutherland and Cohen 1994). Abnormal activation of GSK-3β signaling pathways is implicated in the etiology of psychiatric disease; inhibition of the kinase has been proposed to be of therapeutic benefit in psychosis and bipolar diseases (Nakazawa et al. 2001; Beaulieu et al. 2009; Alimohamad et al. 2005; Li et al. 2007). Further, the compound selectively elevated dopamine overflow in rat prefrontal cortex, a biochemical feature shared among antipsychotic medications (Moghaddam and Bunney 1990; Meltzer and Fatemi 1996). It is likely, however, that schizophrenia is not solely regulated by dopamine. It is widely believed that hypoactivity in cortical glutamate pathways is a key feature of the schizophrenic brain (Laruelle et al. 2005). Glutamate neurotransmission, mediated through NMDA-type receptors, is deficient in schizophrenic patients (Javitt 2007). Subanesthetic doses of NMDA receptor antagonists, like ketamine, induce psychotomimetic symptoms in humans (Krystal et al. 1994). Thus, treatments aimed at normalizing glutamate tone, either by increasing glutamate availability (e.g., inhibitors of the glutamate transporter, GlyT-1) or via agonism at certain populations of glutamate receptors (e.g., mGluR2/3 receptor agonists), have efficacy in preclinical screens for antipsychotic activity, though mixed results in the clinic (Moghaddam and Javitt 2012; Field et al. 2012; Schwartz et al. 2012). Therefore, increasing NMDA receptor activity (see also Jardemark et al. 2010) would be expected to reduce psychosis. To this end, ITI-007 increased tyrosine 1472 phosphorylation of mesolimbic GluN2B-type NMDA receptors in vivo, a modification that is known to direct GluN2B subcellular trafficking to plasma membranes, increasing synaptic NMDA activity (Goebel-Goody et al. 2009).

It should be noted, however, that despite the promise of the glutamatergic hypothesis of schizophrenia, no drug that selectively modulates glutamatergic function has yet been successfully developed as an effective antipsychotic. Also, while phosphorylation of GluN2B receptors has been reported to occur after chronic dosing of several current antipsychotic medications to rodents (Hattori et al. 2006; Carty et al. 2012), these same antipsychotics do not improve negative symptoms or cognition clinically. It is likely that a broad spectrum of efficacy across positive, negative, and cognitive symptoms will require a rebalancing of the mesocortical/mesolimbic glutamatergic and dopaminergic systems. Enhancement of mesolimbic GluN2B receptor phosphorylation in combination with enhanced mesocortical dopamine release may provide this balance of activities.

### Activity in a model of depression

Decreased socialization is a core feature of the negative symptoms of schizophrenia that are poorly addressed by existing antipsychotic medications (Tamminga et al. 1998). The compound tested here reduced social avoidance behavior in mice exposed to repeated social aggression. A previous report (Berton et al. 2006) demonstrated that chronic antidepressant treatment (e.g., the SSRI, fluoxetine) attenuated the expression of social defeat behavior of mice in this paradigm. Significantly, neither acute fluoxetine administration nor chronic administration of antianxiety medications (e.g., chlordiazepoxide) replicated the beneficial effect of chronic fluoxetine, implying that the therapeutic effects reflect the delayed efficacy of antidepressant medications, and further, that this model is useful in detecting antidepressant activity of novel compounds (Krishnan and Nestler 2011). The significant binding of ITI-007 to SERT (62 nM *K*_*i*_), and its activity in the social defeat paradigm support a potential antidepressant activity of the compound. Selective D_2_ receptor or 5-HT_2A_ receptor modulators have not been as extensively evaluated as the serotonin reuptake inhibitors in the social defeat model of antidepressant activity. Unopposed dopamine D_2_ receptor antagonism by haloperidol has been shown to worsen social defeat behavior in rats (Rygula et al. 2008). To the extent that mesolimbic/mesocortical dopamine tone is involved in mediating social stress (Chaudhury et al. 2013), however, the mesolimbic/mesocortical dopaminergic modulation displayed by ITI-007 may have additional benefit for antidepressant activity. Moreover, it has been suggested that selective 5-HT_2A_ receptor antagonism may disrupt the development of conditioned social defeat behavior in hamsters (Harvey et al. 2012). Therefore, the 5-HT_2A_ receptor antagonism combined with SERT affinity may contribute to the antidepressant response with ITI-007 observed here in the chronic social defeat model. In other models of antidepressant activity, 5-HT_2A_ receptor antagonism synergizes with SERT inhibition for greater antidepressant efficacy than SERT inhibition alone (Marek et al. 2005). It should be noted, however, that the compound has not been tested for antidepressant activity more traditional models of antidepressant activity, including tail suspension and forced swim. Regardless of the mechanisms underlying the behavioral effects of ITI-007, the combination of antipsychotic and antidepressant-like activities possessed by the compound in animal models merit further evaluation in human clinical testing for potential value in addressing the negative symptoms and affective symptoms of schizophrenia which are poorly addressed by current antipsychotic medications.

### Therapeutic potential of ITI-007

Based on the in vitro and in vivo activities of ITI-007, described here in animals, we hypothesize that in humans, the high potency 5-HT_2A_ receptor blockade possessed by ITI-007 will result in relatively selective 5-HT_2A_ receptor antagonism at low doses and that its unique dopamine receptor activity and serotonin reuptake inhibition may provide additional benefit from the combined pharmacology as the dose is increased. Furthermore, we speculate that this may permit, with increasing dose levels, the “dialing in” of additional dopaminergic and serotonin reuptake inhibition for broad antipsychotic and antidepressant activities with reduced liability for movement disorders. Ultimately, the potential of ITI-007 for the treatment of schizophrenia and other psychiatric and neurologic disorders awaits further study in humans. The compound is currently under investigation in advanced human clinical studies.
